# Supplementary material for: Making consultation meaningful: Insights from a case study of the South African mental health policy consultation process
Source: PLoS One. 2020 Jan 29;15(1):e0228281. doi: 10.1371/journal.pone.0228281 (PMC6988953; doi:10.1371/journal.pone.0228281)
Supplement: S1 Table — (DOCX) [file pone.0228281.s001.docx]

Making consultation meaningful: Insights from the South African mental health policy consultation process

S1 Table: Codes for analysis of draft and final policy documents

| **Code** | **Coding definition** |
| --- | --- |
| Change in wording of draft policy content | The phrase or concept appears in both the draft and final policy documents, but the content of the final policy represents an amended version of the draft policy wording |
| Addition to draft policy content | An additional phrase or concept that appears in the final policy document that did not appear in the draft policy document |
| Deletion of draft policy content | Content that appears in the draft policy but not in the final policy document |
