# Supplementary material for: Making consultation meaningful: Insights from a case study of the South African mental health policy consultation process
Source: PLoS One. 2020 Jan 29;15(1):e0228281. doi: 10.1371/journal.pone.0228281 (PMC6988953; doi:10.1371/journal.pone.0228281)
Supplement: S2 Table — (DOCX) [file pone.0228281.s002.docx]

Making consultation meaningful: Insights from the South African mental health policy consultation process

S2 Table: Codes for analysis of interview data

| **Main theme** | **Sub-themes** |
| --- | --- |
| Knowledge about or involvement in broader policy development process | Pre-summit policy development |
|  | Post-summit policy development |
| Information and consultation transparency about the policy consultation | Pre-summit consultation and information |
|  | Post-summit consultation and information |
| Provincial follow-through | - |
| Impact of consultation summit | Signalled priority |
|  | Influence on policy |
| Perspectives on final policy | General perspectives about the final policy |
|  | Perspectives about the implementation of the policy |
| Opportunities for service-user input | - |
