# Supplementary material for: Making consultation meaningful: Insights from a case study of the South African mental health policy consultation process
Source: PLoS One. 2020 Jan 29;15(1):e0228281. doi: 10.1371/journal.pone.0228281 (PMC6988953; doi:10.1371/journal.pone.0228281)
Supplement: S3 Table — (DOCX) [file pone.0228281.s003.docx]

Making consultation meaningful: Insights from the South African mental health policy consultation process

S3 Table: Codes for analysis of procedural issues at national summit

| **Code** | **Coding definition** |
| --- | --- |
| Awareness of time constraints | All references relating to time and timing in group sessions (e.g. concern about limited time, time available for the different aspects, and timing of breaks etc.) |
| Microphone management | All references relating to the use of microphone within the group sessions (either explicit or indirect) |
| General procedural comments | Any general references to process or procedural issues relating to the consultation process, particularly pertaining to interaction |
| Engagement with draft documents | All explicit references to engaging with the draft policy or draft summit declaration during group discussions |
| Awareness of needing to formulate recommendations | All references during group sessions indicating explicit awareness of needing to formulate and/or capture specific recommendations for feedback |
| Processes for formulating recommendations | All references relating to processes for formulating proposals or recommendations made during group sessions |
