# Supplementary material for: Making consultation meaningful: Insights from a case study of the South African mental health policy consultation process
Source: PLoS One. 2020 Jan 29;15(1):e0228281. doi: 10.1371/journal.pone.0228281 (PMC6988953; doi:10.1371/journal.pone.0228281)
Supplement: Attachment — Submitted filename: Response to Reviewers.doc [file pone.0228281.s004.doc]

Dr Debbie Marais

Faculty of Medicine and Health Sciences

Stellenbosch University

South Africa

Dr Sara Rubinelli

Academic Editor

*PLOS ONE*

28 November 2019

Dear Dr Rubinelli,

Thank you for your prompt feedback and the reviewers’ comments on our manuscript, *“Making consultation meaningful: Insights from the South African mental health policy consultation process”.* We appreciate the feedback from the reviewers and believe that their input has assisted us to improve this paper. Please find our responses to these comments in the table below, with reference to the pages in the manuscript where these changes have been made, in track changes.

Thank you again for your consideration of this manuscript.

Sincerely,

Debbie Marais

Responses to reviewers’ comments

| **Academic Editors’ Comments** | | | |
| --- | --- | --- | --- |
| **#** | **Comment** | **Response** | **Manuscript reference** |
| 1 | Please ensure that your manuscript meets PLOS ONE's style requirements, including those for file naming. | The style guide and file naming requirements have been carefully attended to and the manuscript and files amended accordingly. | Manuscript and supporting files |
| **Comments from Reviewer 1** | | | |
| **#** | **Comment** | **Response** | **Manuscript reference** |
| 1 | Abstract and Methods: the abstract does not include any methods description. But this needs to be included. The abstract should preferably include for example the number and type of documents reviewed. The number of transcribed audio-recordings analysed, and the sampling and number of key informant interviews. As well as the period of data collection and analysis. This type of information that can give the reader a better insight into the research process such as the amount of data analysed and how thorough the process was carried out. This form of summary information could also be given in the methods section, e.g. on line 156, page 7 | This detail has been included in the abstract and in the methods section. | Abstract: p. 2  Methods: pp. 7-10 |
| 2 | Line 186, page 8. The data collection period included the review of documents, the transcription of audio-recordings and key informant interviews? The key informant interview data collection started November 2013 (emails sent out) and ended in December 2013 (line 192, page 9). When did the review of documents and transcription and analysis of audio-recordings start and end? | Detail regarding when each component of the data collection and analysis has been added to the methods section (subsections a-c). | Methods: pp. 7-10 |
| 3 | Methods: line 163-165, page 7. About the audio-recordings. It would be interesting to know a little more about consent in relation to the audio-recorded data. Were conference participants aware of that they were being recorded and that their discussions might be used for research, audit or similar? | The issue of consent for the recording of the summit breakaway sessions has been included in the methods section. | Methods: p. 10 |
| 4 | Methods: line 192, page 9. Who undertook the key informant interviews? | The principal author conducted the interviews. This detail has been included in the methods section. | Methods: p. 9 |
| 5 | Methods: line 219-225, page 10. It would be interesting to know here, in relation to the audio-recordings from the two-day summit, how the time calculations (that is, the descriptive percentages presented in Table 8,10 and 11) were made using the transcribed audio-recording information. This is briefly mentioned elsewhere in the article. However, this could be made clearer and placed so that the information is easy to find for the reader. | Detail regarding time calculations has been added to the methods section. | Methods: pp. 11-12 |
| 6 | Methods: Table 9, page 26. It is stated in the article that the key informant interviews were anonymised. Looking at Table 9 on page 26, there is a name mentioned in the Table; is it correct it the case that the audio-recordings from the two-day summit also were anonymised? In other words, this is a name given by the article authors and not the real name of a summit participant? This should be made clearer. | Thank you for pointing this out. The name that appeared in the table (now Table 11) has been removed. | Results: Table 11: p. 29 |
| **#** | **Comment** | **Response** | **Manuscript reference** |
| 7 | Data analysis, key informants. 1) Table 1 on page 8, outlines the role of the study participants (the key informant interviewees). However, in Table 2-7 names of interviewees are being used. It would, I believe and if possible, be more relevant and interesting to use the key informants’ roles and gender (could e.g. be marked with Role and then “F” and “M” for Female/Male) rather than their names in relation to their quotes. | Thank you for this point, which helped us to make the information included in the table providing participant information (now Table 2) clearer. We have chosen to add the pseudonyms to the table so that this can serve as a reference point for the tables that follow, in which we have kept the pseudonyms to refer to each participant. | Results: Table 2: p. 13 |
| 8 | Data analysis, transcribed audio-recordings from the two-day summit, line 392, page 23. it would be helpful if the authors could add to this section of the article that the information concerning “Time availability in breakaway group sessions” derive from the audio-recordings. | This detail has been added to the results section. | Results: Time availability in breakaway group sessions: p. 26 |
| 9 | Discussion/limitations. Each method used, that is, the review of documents, the audio-tape transcript analysis, and the analysis of the key informant interviews should preferably be discussed in terms of their potential limitations. Whilst this is mentioned in the article, it would be good with a brief paragraph discussing the main limitations, encompassing all methods used, in the discussion part of the article too. Did the authors’ feel that they had been able to analyse all data sufficiently? If not, what would have been good to pay further attention to? What about the gender distribution in the key informant interviews, in what way might this have impacted on data analysed? And so forth. | A summary of limitations has been added to the discussion section. | Discussion: p. 42 |
| 10 | Proof reading. There are some small mistakes here and there in the article. For example, 1) there appear to be a piece of cited text on line 61-62, page 4, but no page number to the reference that the quote is from. The authors should go through the article to ensure that quotes etc. are referenced correctly. 2) The reference list needs a little more attention. For example, reference 2 include information such as “copyright” and “All rights reserved”; reference 4 and 33 do not state which Government the reports concern or in what country the reports were published, and reference 41 has a “*” in its article title, which doesn’t seem to fit. 3) The text in Table 3 on page 13 and 14, has gaps in it (after the word “So” in Table 3 on page 13, and after the word “then” in Table 3 on page 14). | Thank you for pointing out these errors. These have been corrected. (Note: Table 3 is now Table 4) | Introduction: p. 4 line 65  Results: Table 4 pp. 16-17  Discussion: p. 37 line 746  References: pp. 45-52 |
| **Comments from Reviewer 2** | | | |
| **#** | **Comment** | **Response** | **Manuscript reference** |
| 1 | Title: Include within the title that it is a case study | The title has been changed accordingly. | Title page: p. 1 |
| 2 | Abstract: It would improve clarity if the abstract is divided into background, methods, results & conclusion. | The abstract has been divided into these sections. | Abstract: p. 2 |
| 3 | Methods: Details on the task team that finalized the report, or who commissioned this team would be helpful. | This detail has been included in the Context of study section. | Context of study: p. 6 |
| **#** | **Comment** | **Response** | **Manuscript reference** |
| 4 | Methods: Reorganize methods subsections for more clarity and consistency into the 3 phases: a. Policy documents b. Key Informant Interviews: i. Participant Recruitment (Also moving lines 185-187 “The national mental health summit programme was used to identify potential participants. Contact was made telephonically or via e-mail in November 2013 to inform these individuals about the study and explore their willingness to participate.” within this section ii. Data collection c. Provincial and national summit documents and audio. | Thank you for this recommendation which has helped to make the sub-sections and chronology of the methods clearer. The headings have been added and amended accordingly. | Methods: pp. 8-10 |
| 5 | Methods: Data analysis in the third phase is a bit confusing. The focus seems to be on the ten breakaway group discussions from the national summit, however there is no data analysis mentioned for the provincial mental summit reports and transcripts. | Clarity regarding the provincial reports has been added to the methods section (sub-section c).  The heading of the relevant section in the analysis section (sub-section c – Third phase…) has been amended to make the focus on the national transcripts clearer. | Methods: p. 10  Results: p. 11 |
| 6 | Methods: Identify interviews as “semi-structured interviews”, since an interview schedule was used. | This detail has been added to the methods section (sub-section b). | Methods: p. 9 |
| 7 | Methods: From your results, it seemed that you also performed some triangulation between different methods (“this was partially confirmed by observations that the policy document did not appear to change substantively”) – it would be helpful to mention at the end of your data analysis section whether there was any effort in comparing or triangulating between different methods, as this is often done in case studies. | Triangulation was conducted across the results from the different phases of analysis. However, this was done as a means of showing areas of convergence and divergence with respect to questions relating to a theoretical framework that was used as a lens to make sense of the movement of different kinds of knowledge through the process. This theoretical paper is in process. We believe that the detail on triangulation is thus beyond the scope of the current paper. | No change |
| 8 | Results: The number of participants in the key informant interviews and characteristics (Table 1: Participant information) should be included in the results instead of methods. | Participant information has been moved to the results section. | Results: p. 13 |
| 9 | Results: Phase 2 includes a variety of sub-sections (i.e. Impact of national consultation summit, Gaps in follow-through from provincial to national summits..). An introduction to how these were derived would be helpful – were these based on the interview schedule? Are these all themes (in which case, language within each of these “themes” should be consistent). | This clarification has been added to the results section. | Results: pp. 13-14. |
| **#** | **Comment** | **Response** | **Manuscript reference** |
| 10 | Results: On line 258, “Two main themes” are mentioned. I would move (see Table 2) to the next line, as those 3 sub-themes arise from Theme 1. Additionally, in labeling Tables 2 and 3, it would be helpful to have consistent language as in the results section (i.e. Table 2: Sub-themes from Theme 1: Purpose of national consultation summit; Table 3: Sub-themes from Theme 2: How the national consultation summit informed policy”). | These amendments have been made to the text and to the tables (now Table 3 and Table 4). | Results: pp. 14-16 |
| 11 | Results: The sub-themes within “gaps in follow-through from provincial to national summits” (Table 4) overlap with one another (i.e. Lack of information about whether and how provincial recommendations transferred to national summit + Provincial feedback at national summit was not consistent or visible enough). Perhaps these are different, but if there is a way to use consistent language throughout the sub-themes, it would help with clarity. Since all these sub-themes were discussing the transfer of information from provincial to national summits, the sub-themes could be reworded to remove redundancy. (e.g. Incomplete or non-systematic transfer of information, Minimal space for feedback, Dependent on individual participants, Unsure about extent of information transfer). | The sub-theme labels in the table (now Table 5) have been amended to make the content to which each sub-theme refers clearer and more distinguishable. | Results: Table 5: p. 18 |
| 12 | Results: Include “Phase 3” with the Insights into procedural issues at the national summit sub-title. | The sub-heading has been changed accordingly. | Results: p. 25 |
| 13 | Results: To improve readability of the results in phase 3, you can include 1 or 2 interesting examples of “awareness of time” comments, and just leave in the quantitative % in Table 8. Additionally, the breakaway group topics could be included within the results text of Phase 3. | Thank you for this suggestion. We have decided to leave the table as is, with examples of quotes from each group corresponding to the percentages, as we feel that the quotes give context to the quantitative data.  We have added in a table (Table 9) showing the themes per breakaway group, which will hopefully provide a more user-friendly reference for the reader. | Results: Table 10: no change  Results: Table 9: p. 26 |
| 14 | Results: Table 11 seems to be missing text in the column header (Awareness of need to formulate). | The column heading in the table (now Table 13) has been corrected. | Results: Table 13: p. 33 |
| 15 | Results: For tables 11 and 12, it would be helpful to report on the number of "direct engagement" vs "recommendations formulated by chair" and “silent rapporteurs” vs active rapporteurs” instead of a list of the 10 breakout groups. Since the purpose of your case study is to identify the underlying methods or mechanisms of the consultation process (and not a comparison between the 10 groups) you could do some further grouping in these 2 tables to identify the different strategies or processes used. | Thank you for this suggestion. For consistency we have chosen to keep the structure of the tables (now Tables 12 and 13) the same. The aforementioned triangulation and theoretical analysis will draw to some extent on the group detail, so we would prefer to keep this detail included.  However, we have included totals in the tables and discussed these in the text, so that the overall proportion of time spent across all groups is clearer. | Results: pp. 30 – 31 (Table 12)  Results: pp. 32 – 34 (Table 13) |
| **#** | **Comment** | **Response** | **Manuscript reference** |
| 16 | Discussion: The concepts of “forward movement” and “backward movement” of inputs is introduced in the discussion – these would be helpful concepts to introduce in the introduction or methods. | These concepts have been included in the methods section. | Methods: p. 7 |
| 17 | Discussion: A breadth of methodological limitations are reported throughout the paper (i.e. inconsistencies in recording and availability, transparency; lack of representation from the DoH/ provinces, poor quality audio recording) – it would be helpful to summarize these in the discussion to identify any limitations with answering your research questions. | A summary of limitations has been added to the discussion section. | Discussion: p. 42 |
| 18 | Minor grammatical: Line 90: change “available knowledges” to available knowledge | Edit has been made. | Introduction: p. 5 line 93 |
